# Hepatic Anti-Oxidative Genes *CAT* and *GPX4* Are Epigenetically Modulated by RORγ/NRF2 in Alphacoronavirus-Exposed Piglets

**DOI:** 10.3390/antiox12061305

**Published:** 2023-06-19

**Authors:** Haotian Gu, Yaya Liu, Yahui Zhao, Huan Qu, Yanhua Li, Abdelkareem A. Ahmed, Hao-Yu Liu, Ping Hu, Demin Cai

**Affiliations:** 1 College of Animal Science and Technology, Yangzhou University, Yangzhou 225009, China; htgu1998@outlook.com (H.G.); yyliu1120@outlook.com (Y.L.); YHzhao0114@outlook.com (Y.Z.); hqu1005@outlook.com (H.Q.); haoyu.liu@yzu.edu.cn (H.-Y.L.); 2 College of Veterinary Medicine, Yangzhou University, Yangzhou 225009, China; yanhuali007206@yzu.edu.cn; 3 Biomedical Research Institute, Darfur University College, Nyala 56022, Sudan; aabdallah@buan.ac.bw; 4 International Joint Research Laboratory in Universities of Jiangsu Province of China for Domestic Animal Germplasm Resources and Genetic Improvement, Yangzhou 225009, China

**Keywords:** RORγ, PEDV, *GPX4*, oxidative stress, NRF2, histone modifications

## Abstract

As a member of alpha-coronaviruses, PEDV could lead to severe diarrhea and dehydration in newborn piglets. Given that lipid peroxides in the liver are key mediators of cell proliferation and death, the role and regulation of endogenous lipid peroxide metabolism in response to coronavirus infection need to be illuminated. The enzymatic activities of SOD, CAT, mitochondrial complex-I, complex-III, and complex-V, along with the glutathione and ATP contents, were significantly decreased in the liver of PEDV piglets. In contrast, the lipid peroxidation biomarkers, malondialdehyde, and ROS were markedly elevated. Moreover, we found that the peroxisome metabolism was inhibited by the PEDV infection using transcriptome analysis. These down-regulated anti-oxidative genes, including *GPX4*, *CAT*, *SOD1*, *SOD2*, *GCLC*, and *SLC7A11*, were further validated by qRT-PCR and immunoblotting. Because the nuclear receptor RORγ-driven MVA pathway is critical for LPO, we provided new evidence that RORγ also controlled the genes *CAT* and *GPX4* involved in peroxisome metabolism in the PEDV piglets. We found that RORγ directly binds to these two genes using ChIP-seq and ChIP-qPCR analysis, where PEDV strongly repressed the binding enrichments. The occupancies of histone active marks such as H3K9/27ac and H3K4me1/2, together with active co-factor p300 and polymerase II at the locus of *CAT* and *GPX4*, were significantly decreased. Importantly, PEDV infection disrupted the physical association between RORγ and NRF2, facilitating the down-regulation of the *CAT* and *GPX4* genes at the transcriptional levels. RORγ is a potential factor in modulating the *CAT* and *GPX4* gene expressions in the liver of PEDV piglets by interacting with NRF2 and histone modifications.

## 1. Introduction

Coronaviruses (CoVs) of RNA viruses seriously affect the health of humans, bats, livestock, and many other wild animals. CoVs infection could harm these animals’ gastrointestinal, respiratory, liver, and central nervous systems [1,2,3]. Approximately 70% of the emerging pathogens which could infect humans are zoonotic [4]. In particular, RNA viruses are more prone to mutate than other microorganisms and could cause significant outbreaks due to unique genetic changes [5]. In addition, virus transmission from the natural wild animal hosts to humans can be implemented with the help of domestic animals as the intermediate hosts [6,7]. Therefore, the spread of CoVs in livestock is also worthy of attention. Porcine epidemic diarrhea virus (PEDV) is a member of alpha-CoVs in the family coronaviridae. It possesses a genome consisting of a single positive strand of RNA and could lead the newborn piglets to severe diarrhea, vomiting, and dehydration [8,9]. While PEDV could infect pigs of all ages, the mortality rate of newborns during PEDV infection can be up to 100% [10]. The feces and vomitus from the infected pigs could transmit the virus to healthy pigs, and it is the main route of transmission of PEDV, which is also the reason for the high infectivity of PEDV [11]. The persistence of PEDV causes great losses to the pig industry and is a menace to human security. The pathogenesis of CoVs is closely related to oxidative stress (OS). A defective redox balance during infection is associated with viral activation of pro-inflammatory cytokine responses and the triggering of cell death due to reactive oxygen species (ROS) overload [12]. The imbalance between ROS generation and anti-oxidant defense efficiency always induces OS [13]. The enhanced generation of ROS will lead to lipid peroxidation (LPO), oxidize protein, and activate the programmed cell death (PCD) pathway [14,15]. Moreover, while infected by the virus, OS is the main pathogenic mechanism for inflammation or tissue injury [16,17]. ROS are oxygenated by-products from the utilization of oxygen in the mitochondrial energy metabolism and are indispensable in cellular homeostasis [18]. The anti-oxidant enzymes and scavengers are the natural defense system against ROS, mainly including superoxide dismutase (SOD), catalase (*CAT*), glutathione synthetase (*GSS*), glutathione peroxidase (GPX), and glutathione (GSH) [19]. The excess free radicals could be removed by anti-oxidant enzymes, and the main mechanism is that SOD and *CAT* convert hydrogen peroxide to water and oxygen [20]. GPX is a group of enzymes containing selenium, hydrogen peroxide, and organic peroxides that can be catalyzed to alcohol [21]. *GPX4* is the only enzyme that can remove LPO on biofilms, while GSH is used as a substrate to reduce LPO [22]. It is worth emphasizing that the biosynthesis of *GPX4* is regulated by the mevalonate (MVA) pathway. MVA produces isopentenyl PPi (IPP) to modulate the expression of *GPX4* through prenylation [23,24]. Notably, MVA is critical for cholesterol production, while the high cholesterol levels in the cell membrane have been suggested to increase the risk of CoVs entry by enhancing the virus-binding activity and facilitating membrane fusion [5,25]. It is well-known that sterol regulatory element-binding protein 2 (SREBP2) can bind directly to the promoters of the key genes involved in MVA biosynthesis, so it is the primary transcriptional factor of MVA metabolism [26]. Importantly, as a member of the retinoid-related orphan nuclear receptors, retinoic acid receptor-related orphan receptor gamma (RORγ) can drive the MVA pathway over SREBP2 by epigenetic regulations, which has been shown in our previous study [27]. Therefore, RORγ can potentially affect LPO by targeting the MVA pathway during virus infection. Moreover, given the fact that the primary transcription factor in modifying LPO and OS is nuclear factor erythroid 2-related factor 2 (NRF2), whether there is interactive communication between RORγ and NRF2 needs to be illuminated.

The liver is the primary site for cholesterol production from MVA. It is notable that CoVs can also impair the digestive and endocrine systems [28,29], while numerous cases of liver injury are reported in patients infected with CoVs. Immune cell-mediated injury, ischemia, hypoxia, and hepatotoxicity have been suggested [30]. However, the potential mechanisms are still unknown. In this study, we aimed to reveal the underlying mechanisms of lipid peroxide regulation in the liver of piglets exposed to PEDV. The results showed that PEDV infection led to OS by strongly inhibiting anti-oxidation processes including enzyme activities and gene expressions. The down-regulated genes *CAT* and *GPX4* were attributed to the transcriptional modulation of key factors RORγ and NRF2 alone or in combination. In addition, we also found that the epigenetic marks H3K4me1/2/3 and H3K9/27ac and co-factor p300 were involved in the regulation by facilitating the transcriptional repression with the action of RNA polymerase II.

## 2. Materials and Methods

### 2.1. Animals

The animals used for experiments were Large White piglets (~15 kg). The piglets were naturally infected with PEDV and were selected as the experimental samples from a pig farm with a PEDV outbreak. The animals used in this study were all seven-day-old male piglets, and the piglets chosen as the PEDV group all had clinicopathological features of porcine epidemic diarrhea such as vomiting, diarrhea, dehydration, and watery diarrhea. Moreover, the piglets were confirmed to be infected with PEDV by qPCR in our previous study [29]. In comparison, seven uninfected piglets (~15 kg) were used as a negative control as described. The IACUC of Jiangsu Province (SYXK (Su) IACUC 2012-0029) reviewed and approved all animals for experiments. The piglets were euthanized humanely to collect their liver tissues; before that, their raising conditions were the same. Liquid nitrogen was used to snap-freeze acquired tissues until analysis, and tissues were stored at −80 °C.

### 2.2. RNA-Seq Analysis

Three samples were randomly selected from two groups for RNA-seq sequencing. Cold PBS was used to wash the liver tissues randomly selected from the PEDV-infected and uninfected piglets. In order to prepare the total RNA for RNA-seq, Illumina Tru-Seq RNA Sample Preparation Kit was used as previously described [31]. Firstly, the quality of libraries should be eligible, and an Agilent 2100 Bioanalyzer was used to check it. After that, an Illumina HiSeq 2000 sequencer (BGI Tech, Wuhan, China) was used to carry out the high-through sequencing. As in the previous description, the standard BWA–Bowtie–Cufflinks workflow was used to analyze the sequence data [27]. In short, BWA and Bowtie software were used to map sequence reads to the susScr3 assembly. The transcript assembly, quantification of the normalized gene, isoform expression, as well as analysis of differentially expressed genes were all performed using the Cufflinks package. The GSEA tool (GSEA v.3.0) was used for the Gene Set Enrichment Analysis while DAVID Bioinformatics Resources 6.8. was used for gene ontology analysis.

### 2.3. qRT-PCR Analysis

TRIzol Reagent (Invitrogen, 15596026, Waltham, MA, USA) and HiScript II Q RT SuperMix (Vazyme biotech, R222-01, Nanjing, China) were used to extract the RNA and for reverse transcription, respectively. Then, AceQ qPCR SYBR Green Master Mix (Vazyme Biotech, Q111-02, Nanjing, China) and an ABI QuantStudio 3 Real-Time PCR Instrument were used for qRT-PCR analysis. Glyceraldehyde-3-Phosphate Dehydrogenase (*GAPDH*) was used as an internal reference. The ^−ΔΔCt^ method was used to analyze the gene expression of indicated genes. The primers are shown in Table 1.

### 2.4. Western Blotting Analysis

Phosphatase and protease inhibitors were added to cell lysis buffer (Biosharp, BL509A Hefei, China), and then the mixed solution was used to homogenize liver tissues. The protein of samples was adjusted to equal amounts, and then they were separated in 10% SDS-PAGE gels. After being transferred to PVDF membranes (Millipore, IPVH00010, Burlington, CA, USA), 5% skimmed milk was used to block samples for 1 h. Membranes were incubated with specific primary antibodies at 4 °C for 12 h before being incubated with HRP-conjugated secondary antibodies. Finally, a high-sensitivity ECL kit (NCM Biotech, P2300, Suzhou, China) and Tanon 5200 Multi imaging system were used for chemiluminescence detection. The antibodies used for Western blotting are shown in Table 2. These antibodies for testing porcine tissues have been suggested at the instruction of the manufacturer and have been validated in our and other previous studies [32,33].

### 2.5. ROS Levels Assay

Firstly, the tissues were homogenized and resuspended in PBS and then the supernatant was collected. The OxiSelect In Vitro ROS/RNS Assay Kit (Cell Biolabs, STA-347, San Diego, CA, USA) was used for measurement. The non-fluorescence probe of DCFH-DiOxyQ was primed to the highly reactive DCFH. The highly fluorescent DCF from DCFH oxidized by ROS could be read at 480 nm excitation and 530 nm emission to measure the levels of ROS in liver tissues.

### 2.6. Determination of SOD and CAT

The Total Superoxide Dismutase Assay Kit with WST-8 (Beyotime, S0101S, Shanghai, China) and Catalase Assay Kit (Beyotime, S0051, Shanghai, China) were used to determine the enzyme activities of SOD and *CAT*. The absorbances of SOD and *CAT* were measured at 450 nm and 520 nm, the contents were calculated according to the standard curve, and then they were standardized to protein concentrations.

### 2.7. Measurement of GSH and MDA

GSH and GSSG Assay Kits (Beyotime, S0053, Shanghai, China) were used to measure the content of GSH, and then they were standardized to tissue weight. With the indicated treatments, the LPO MDA Assay Kit (Beyotime, S0131S, Shanghai, China) was used to determine the MDA contents. Briefly, the samples were centrifuged after being treated with lysis buffer, then supernatants were mixed with TBA detection solution to measure the absorbance at 532 nm, and then the contents of MDA were calculated according to the standard curve. Finally, the data were standardized to tissue weight.

### 2.8. Hepatic Complexes I, III, V Activities and ATP Content Assay

As in a previous description, respective commercial assay kits (Comin Technologies, Co., Ltd., Suzhou, China) were used to measure the activities of mitochondrial respiratory chain complexes I, III, and V [34]. The ATP Assay Kit (Beyotime, S0026, Shanghai, China) was used to determine the content of liver ATP.

### 2.9. ChIP-qPCR Analysis

As in a previous description [31,35], ChIP-qPCR was performed with the following modifications. In short, 1% formaldehyde was used to cross-link liver tissues for 5 min, then the fixing buffer containing 100 mmol/L NaCl, 50 mmol/L Hepes-KOH, 1 mmol/L EDTA, and 0.5 mol/L EGTA was used for pulverizing and resuspending. After that, glycine was used to quench samples for 6 min on ice. Then, 140 mmol/L NaCl, 50 mmol/L HEPES pH 8.0, 10% glycerol, 1 mmol/L EDTA, 0.5% NP40, and 0.25% Triton X-100 were mixed to be the lysis buffer for resuspending the collected precipitation. Next, 200 mmol/L NaCl, 1 mmol/L EDTA, 0.5 mmol/L EGTA, and 10 mmol/L Tris pH 8.0 were mixed to be the washing buffer while 1 mmol/L EDTA, 10 mmol/L Tris HCl pH 8, and 0.1% SDS were mixed to be the shearing buffer; they were used for washing and resuspending the precipitation, and Covaris M220 was used to sonicate the samples. Chromatin fragments were treated with gene-specific antibodies and protein G-conjugated beads for precipitation. Finally, purified ChIP DNA was obtained from samples and RNase A and proteinase K were added, which was then used for qPCR analysis. The primers for ChIP-qPCR are showed in the Table 3.

### 2.10. Analysis of ChIP-Seq

As in a previous description [35], the ChIP-seq analysis was performed with the following modifications. The pipeline of AQUAS Transcription Factor and Histone was used to process fastq files from ChIP-seq. In short, BWA 0.7.1551 was used to map sequencing tags against the Sus Scrofa (pig) reference genome (susScr3). Peak calling was carried out by model-based analysis for ChIP-Seq (MACS; 2.1.0) measurement to identify the enrichment regions over the background using uniquely mapped tags which were filtered and deduped. MACS2, UCSC tools, and bedTools were used to build normalized genome-wide signal-coverage tracks from the original read alignment files. Next, deepTools and HOMER were used to achieve the avg profile and heatmap of the ChIP-seq signal at enriched genomic regions and identified peak-associated genes. Whether a peak was at the transcription start site, transcription termination site, Exon (Coding), 5′ UTR Exon, 3′ UTR Exon, Intronic, or Intergenic was taken as further annotation information.

### 2.11. ChIP-re-ChIP Assays

As in a previous description [31,36], the chromatin immunoprecipitation was performed with the following modifications. To remove rough chromatin solutions from liver tissues, magnetic beads (Thermofisher Scientific, 10004D, Waltham, MA, USA) were first treated with immune serum for 2 h at 4 °C, followed by binding. Then, the indicated antibodies were used for incubating the pre-cleared chromatin solutions at 4 °C overnight. After that, BSA was used to pre-block protein A beads and sonicated salmon sperm DNA was added to precipitate samples. Previous ChIP products obtained immunoprecipitated complexes successfully: eluting them with dithiothreitol (20 mM) at 37 °C for 0.5 h, brief vortexing, and diluting them 50 times were necessary during the procedure of re-ChIP before it centrifuged to clear. Indicated antibodies for secondary ChIP were used to incubate samples overnight at 4 °C, and then qRT-PCR was used to analyze the ChIP-ed DNA.

### 2.12. Statistical Analysis

Two-tailed Student’s *t*-test was used to compare two groups, while one-way analysis of variance (ANOVA) with Tukey’s post hoc test was used for all groups. *p* < 0.05 was considered statistically significant. All data were presented as mean ± SEM. Figures were processed via GraphPad Prism 8.0.

## 3. Results

### 3.1. PEDV Induces Oxidative Stress in the Livers of Piglets

**Figure 1:**
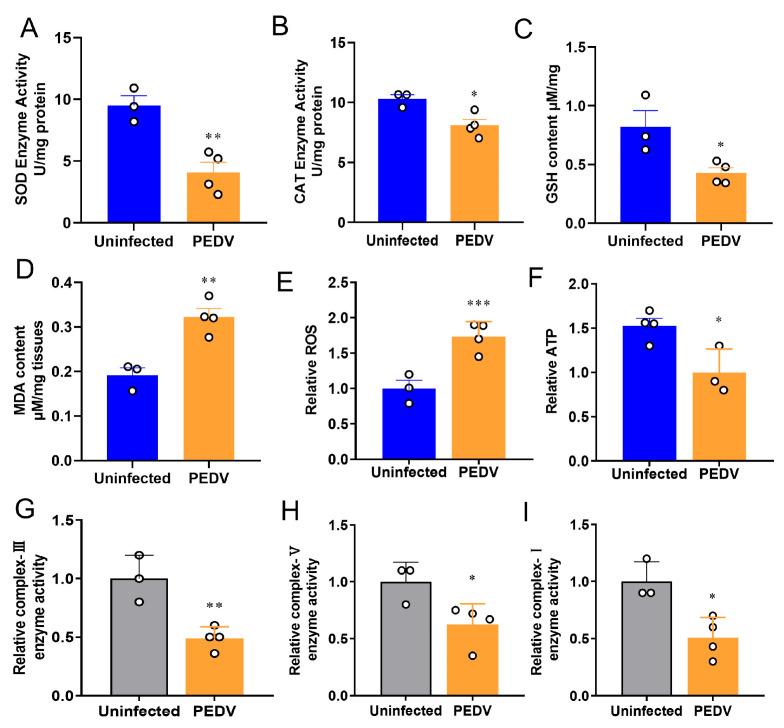
Oxidative stress was observed after being infected by PEDV. (**A**,**B**) The activities of anti–oxidant enzymes SOD and CAT were analyzed and normalized to tissue weight. (**C**,**D**) The contents of GSH and MDA were analyzed and normalized to tissue weight. (**E**) The relative ROS in the PEDV–infected piglets were analyzed. (**F**–**I**) The relative parameters of mitochondria ATP, enzyme complex–I, complex–III, and complex–V were analyzed. (*) *p* < 0.05, (**) *p* < 0.01 and (***) *p* < 0.001 compared with the uninfected sample. The circles represent distribution of results for different samples.

To confirm the occurrence of OS in the liver, we measured the key enzymes previously identified to be altered during PEDV infection [29]. The results showed the anti-oxidative enzymes such as SOD and *CAT* were reduced in the PEDV piglets significantly (Figure 1A,B). Accordingly, decreased GSH and increased MDA levels were observed in these piglets when compared to the uninfected (Figure 1C,D). Moreover, the results showed that PEDV led to a higher ROS production in the piglets exposed to PEDV (Figure 1E). Additionally, we demonstrated that PEDV exposure remarkably diminished the enzyme activities of mitochondrial complexes I, III, and V and the content of ATP (Figure 1F–I). Together, these data reveal that PEDV entry caused the leading OS in the livers of piglets.

### 3.2. Anti-Oxidative Genes Are Susceptible to PEDV Infection

**Figure 2:**
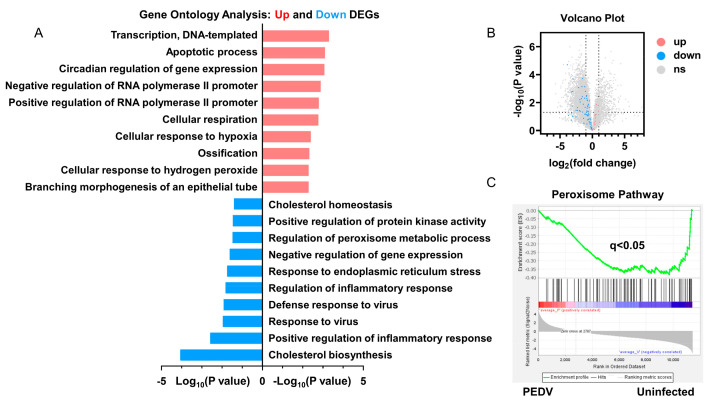
The transcriptional profiling showed that the anti–oxidative pathway was modulated by PEDV infection. (**A**) Gene ontology (GO) analysis showed that the anti–oxidative metabolic process is one of the most enriched pathways. (**B**) Volcano plot for the hepatic transcriptome of anti–oxidation measured by RNA–seq analysis. (**C**) The GSEA plot of the differentially expressed genes in the anti–oxidative pathway of the two groups. FDR, false–discovery rate.

**Figure 3:**
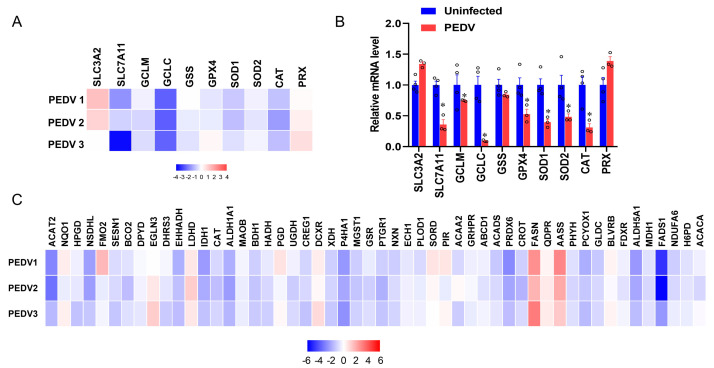
The expressions of genes included in anti–oxidation and fatty acid oxidation are inhibited by PEDV. (**A**) Heatmap of mRNA expression changes of the anti–oxidant metabolism genes from the data of RNA–seq. (**B**) The qRT–PCR analysis confirmed the mRNA expression changes of the anti–oxidant metabolism genes after being infected by PEDV. (**C**) Heatmap of mRNA expression changes of the fatty acid oxidation genes from the data of RNA–seq. (*) *p* < 0.05 compared with the uninfected sample. The circles represent distribution of results for different samples.

RNA-seq analysis was performed to delineate the effects of PEDV infection on the core transcription programs of livers. After being infected by PEDV, the 1500 most down-regulated transcripts were selected for gene ontology analysis, and we found that the regulation of the peroxisome metabolic process was one of the most enriched pathways (Figure 2A). Moreover, a volcano plot was conducted to visualize the differentially expressed genes between the two groups. We observed that the down-regulated genes were more than up-regulated genes for anti-oxidant pathways after being infected by PEDV (Figure 2B). In association with these results, using GSEA, we found that PEDV infection suppressed the hallmarks of the anti-oxidation pathway strongly (Figure 2C). Next, we performed the pathway-focused data analysis, underlining the signaling of GSH and anti-oxidation enzymes against OS. The results showed that the key anti-oxidation genes including *SLC7A11*, *GCLM*, *GCLC*, *GSS*, *GXP4*, *SOD1*, *SOD2*, and *CAT* were down-regulated after being infected by PEDV (Figure 3A). The qRT-PCR analysis was used to further validate these decreased transcripts (Figure 3B). However, *SLC3A2* and *PRX* gene expressions were not significantly changed in response to PEDV exposure as shown in Figure 3A,B. Notably, PEDV infection inhibited the majority of genes enrolled in the lipid oxidation pathway at the transcriptional level significantly, providing further evidence of OS (Figure 3C). Collectively, these results indicate that the anti-oxidative metabolic pathways are susceptible to PEDV infection and may be the critical contributor to OS in the liver of infected piglets.

### 3.3. RORγ and NRF2 Are Potential Modulators to Control CAT and GPX4

**Figure 4:**
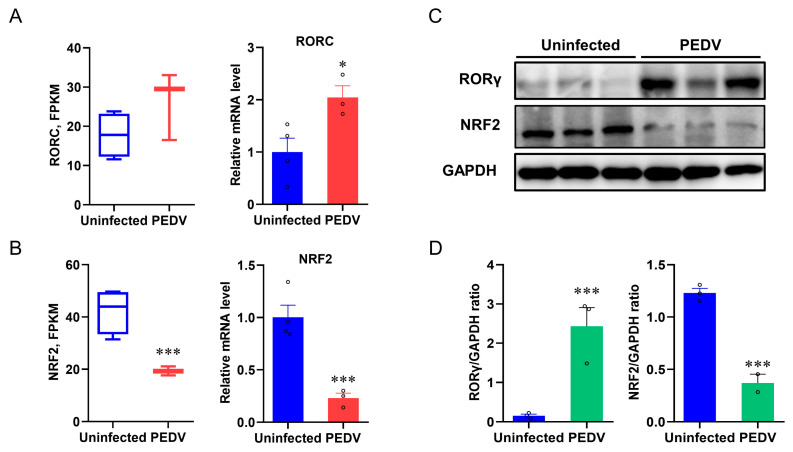
The expressions of RORγ and NRF2 in livers were dysregulated after being infected by PEDV. (**A**,**B**) Fragments Per Kilobase of transcript per Million mapped reads of RORC (encoding RORγ) and NRF2 were measured by RNA–seq and the mRNA expressions were measured by qRT–PCR. (**C**,**D**) The expression of RORγ and NRF2 at the protein level was evaluated via Western blotting. The intensity of these two proteins was normalized to GAPDH protein contents. (*) *p* < 0.05 and (***) *p* < 0.001 compared with the uninfected sample. The circles represent distribution of results for different samples.

Based on the PEDV-induced OS and altered peroxisome pathway results, we sought to identify the key transcriptional factors that potentially control these events. The expression of RORγ was studied because it modulates the MVA pathway and LPO [37]. We found an incoordination between the expression of RORγ and anti-oxidative genes from the results of RNA-seq analysis and qRT-PCR, which showed that PEDV infection strongly up-regulated the mRNA expression of RORγ (Figure 4A) and the immunoblotting validated the increased RORγ expression at the protein level (Figure 4C,D). NRF2 is the key factor of LPO modulation, and we determined that the mRNA and protein expression of NRF2 were strongly down-regulated in response to PEDV infection (Figure 4B–D), suggesting it may be important for the regulation of the anti-oxidative pathway.

### 3.4. RORγ and NRF2 Transcriptional Binding at CAT and GPX4 Were Reduced

**Figure 5:**
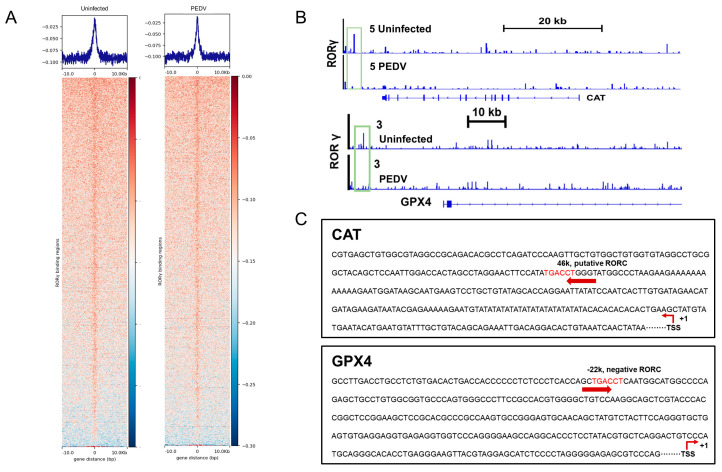
RORγ binding occupancies were reduced in the PEDV piglets. (**A**) ChIP–seq profiles (top) and heatmaps of ChIP–seq signal intensity (bottom) of RORγ within ±10 kb windows around the center of peak regions. (**B**) ChIP–seq signal visualization of RORγ at representative anti–oxidant metabolism genes *CAT* and *GPX4*, the numbers coupled with the group names of Uninfected or PEDV represent the maximum range of data. (**C**) Schematic diagram depicting the putative RORE sequence on the enhancers of *CAT* and *GPX4*.

**Figure 6:**
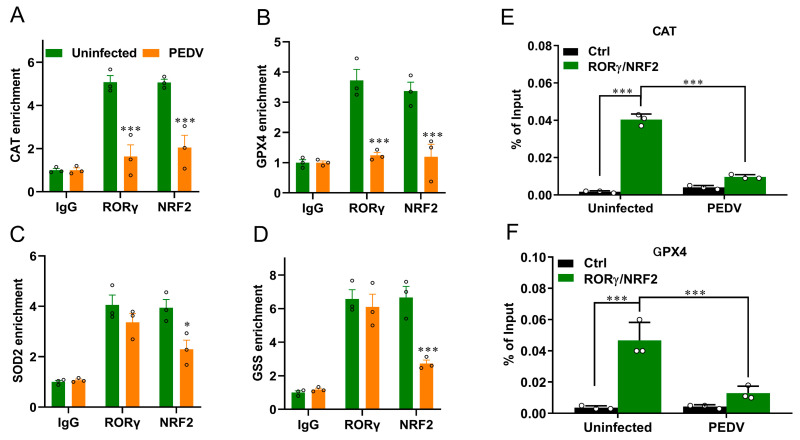
PEDV infection decreased the enrichment of RORγ and NRF2 at target loci of *CAT* and *GPX4* genes and their physical interaction. (**A**–**D**) ChIP–qPCR analyses of RORγ and NRF2 occupancies at the locus of *CAT*, *GPX4*, *SOD2*, and *GSS*, normalized to IgG. (**E**,**F**) ChIP–re–ChIP analysis of the combined binding of RORγ and NRF2 at the locus of *CAT* and *GPX4*. (*) *p* < 0.05 and (***) *p* < 0.001 compared with the uninfected sample. The circles represent distribution of results for different samples.

We next performed a ChIP-seq analysis and demonstrated that RORγ could directly bind to the core genes *CAT* and *GPX4* involved in anti-oxidation (Figure 5A,B). Intriguingly, we found that PEDV infection has no remarkable effects on the genome-wide binding of RORγ compared to that of the uninfected (Figure 5A). However, at the enhancers of the specific targets *CAT* and *GPX4*, the binding of RORγ was reduced substantially but not for *SOD2* or *GSS*. Following, a ChIP-qPCR analysis to validate the reduced occupancies at the enhancers of *CAT* and *GPX4* genes (Figure 6A–D). Importantly, in concomitance with the loss of RORγ enrichments, NRF2 binding occupancies were significantly diminished at the same locus of the *CAT* and *GPX4* genes, along with the genes *SOD2* and *GSS* (Figure 6C,D). Therefore, we performed a ChIP-re-ChIP analysis in the livers to examine whether RORγ co-occupies the binding region with NRF2. In the first ChIP, RORγ-chromatin complexes were enriched with specific anti-RORγ antibodies. Using these samples for the second ChIP, a strong NRF2 interaction was measured via the anti-NRF2 antibody at the enhancers of the *CAT* (Figure 6E) and *GPX4* (Figure 6F) genes. We observed that PEDV exposure dramatically reduced the interacted occupancies in the livers, implying that RORγ binds concurrently with NRF2 to the core anti-oxidative genes.

### 3.5. Histone Modifications Facilitate the Transcriptional Suppression of GPX4 and CAT

**Figure 7:**
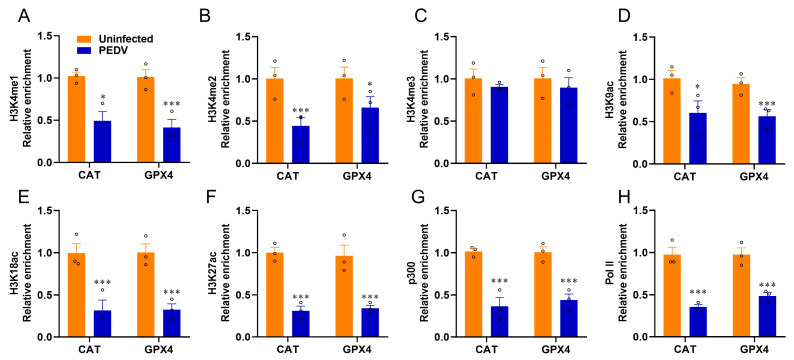
PEDV infection modifies histone modification at the locus of *CAT* and *GPX4*. (**A**–**F**) The relative enrichment of histone marks’ (H3K4me1/2/3, H3K9ac, H3K18ac, H3K27ac) occupancy was analyzed by ChIP–qPCR. (**G**,**H**) The relative enrichment of co–activator p300 and RNA polymerase II at the locus of *CAT* and *GPX4*. (*) *p* < 0.05 and (***) *p* < 0.001 compared with the uninfected sample. The circles represent distribution of results for different samples.

Given that epigenetic regulations are enrolled in the modulations of nuclear receptors, we used ChIP-qPCR to determine the histone mark enrichments at the *CAT* and *GPX4* genes. The histone marks H3K4me1, H3K4me2, H3K9ac, H3K18ac, and H3K27ac related to transcriptional activation were significantly reduced by PEDV infection, but not H3K4me3, at the enhancers of *CAT* and *GPX4*, respectively (Figure 7A–F). The recruitment of active co-factor p300 and the RNA polymerase II (Pol-II) to the target enhancers were also observed to be markedly reduced by PEDV (Figure 7G,H). These data revealed that histone modifications and co-factors played crucial roles in regulating *CAT* and *GPX4* in the piglets exposed to PEDV infection.

## 4. Discussion

CoVs outbreaks and influenza epidemics in recent decades have been a severe threat to global health worldwide [38]. The viral infections result in serious respiratory syndromes and related complications, including liver lesions or renal failure caused by systemic and partial peroxidation responses [39]. The liver plays an important role in mammalian lipid synthesis, oxidation, and metabolism. It is also susceptible to CoVs infection and the virus-activated LPO process [40]. As a member of CoVs, PEDV has significant epidemiology, and the morbidity of piglets is high, while the mortality of that could be up to 100% [41]. Although diarrhea and vomiting are the typical clinical symptoms of PEDV infection [42], liver failure caused by PEDV is also brought to attention. To date, the main preventive and control strategies for PEDV infection are enhancing biosecurity or vaccination, and new anti-PEDV strategies are under intensive exploration [43,44]. In this regard, we provided new findings on the molecular mechanisms of hepatic peroxidation regulation in the PEDV-exposed piglets.

LPO has been suggested to be a hallmark of the severity of CoVs, in which OS acts as a key player in CoVs pathogenesis because of its vital functions during virus entry [45]. Because a redox imbalance has been tightly linked to viral pathogenesis, OS significantly provokes serious cell death as reported [46]. Recently, Marta Martín-Fernandez et al. demonstrated that higher LPO levels are closely related to a higher risk of death when patients were infected with COVID-19 by determining OS status [47]. As a biological free radical chain reaction, LPO could impair cell membranes by inducing membrane damage and functional absorbability [48]. Thus, we performed a complete OS profile evaluation highlighting anti-oxidant enzyme, genes, protein expression, and key metabolic products to demonstrate the roles of peroxidation during PEDV infection, as the biomarkers of LPO, MDA, and 4-HNE are always used for measuring it [49]. Notably, MDA is an indicator of a form of regulated cell death characterized by iron-dependent LPO [49]. We demonstrated that PEDV obviously increased the content of MDA and ROS levels in the live tissues. This is the first evidence to show that the PEDV caused OS induction in the liver lesion via peroxides accumulation. Mitochondrial metabolism is critical for LPO and CoVs entry [50]. Then disrupted by viral infections, the virus-induced mitochondrial DNA (mtDNA) entering the host cells will be enhanced. We have measured the key enzymes involved in the electron transfer chain, and the markedly reduced activities demonstrated the impairments of PEDV in the oxygen-consuming processes. Consistently, specific complex I and III enzyme activities were suggested to contribute to mitochondrial respiration in response to CoVs infection [51]. Given that FASN is critical for lipogenesis, the increased FASN facilitates lipid generation and then accelerates the virus entry through lipid rafts [52]. Lipid rafts act as sub-domains of the plasma membrane and provide the platforms for viral endocytosis [53]. Therefore, further studies need to explore whether FASN is another crucial target that benefits anti-PEDV infection.

As aforementioned, we also found the reduced content of GSH along with the raised MDA in the livers. Indeed, GSH has been demonstrated to defend CoVs by inhibiting the inflammatory response [54]. The changed GSH must have resulted from the descended expressions of crucial enzymes involved in the anti-oxidative pathway. The extracellular cystine and intracellular glutamate exchange across the plasma membrane to synthesize the GSH via the system Xc^−^ [55,56]. It has been reported that the expression of *SLC7A11* would positively regulate the activity of the Xc^−^ antiporter as the system Xc^−^ has a subunit uniquely encoded by *SLC7A11* [57]. The down-regulated *SLC7A11* would blunt the exchange processes to diminish glutamine production, while the reduction in *GCLC* and *GCLM* further triggered the failure of GSH synthesis [58]. The inhibition of GSH synthesis might be a key event for LPO in the livers of piglets, and the depletion of anti-oxidation enzymes must be another issue.

It is worth mentioning that SOD members are potential predictors of CoVs progression [59]. Again, *CAT* was considered to be a potential therapeutic for CoVs via modulating cytokine production, oxidative injury protection, and replication repression [60]. In line with this notion, PEDV-exposed piglets showed a significant down-regulation of *CAT* expression in the liver. Moreover, the strongly decreased expression of lipid oxidation genes observed supported that the PEDV-induced hepatic oxidative injury is due to the disrupted lipid metabolism. *GPX4* activity is an excellent marker for CoVs entry and features obvious prevention to maintain host cells by reducing the multi-cellular hijacking. It is worth noting that *GPX4* is the only enzyme that can remove lipid peroxides from biofilms, and its de novo synthesis is vulnerable to the MVA pathway [61,62]. We previously revealed that the transcripts of key genes included in the MVA pathway, such as *HMGCR*, *HMGCS1*, and *MVK*, are dramatically inhibited in the liver tissues exposed to PEDV [29]. Importantly, we observed that the PEDV infection also inhibited the transcription of *GPX4* and could become one of the dominant factors of LPO during PEDV infection. An interesting finding of the present study is that inhibition did not occur in the RORγ mRNA, even though the protein levels for RORγ were significantly increased. Indeed, RORγ has been proven to bind directly to the genes of the MVA pathway and drive these genes’ transcription [33]. We thus provide the evidence of the drastically reduced RORγ enrichments at the key genes *GPX4* and *CAT*. The ChIP-seq result is pivotal evidence that RORγ directly binds to the core genes involved in the anti-oxidation process. Given that gene regulation is driven by transcriptional binding and initiation, as a transcriptional regulator, the decreased binding enrichment of RORγ contributed to the down-regulated *CAT* and *GPX4* gene expression. Using ChIP-re-ChIP analysis, our data revealed that the NRF2 binding is enriched at the same sites of RORγ at *CAT* and *GPX4* genes, while PEDV could significantly reduce the NRF2 occupancies on these targets using the RORγ-ChIP-ed DNA. This is strong evidence that NRF2 was enrolled in RORγ actions by the reciprocal crosstalk between these two modulators. It has been suggested that pharmacological activation of NRF2 induces a multifaceted anti-inflammatory response to fight against the virus [63]. Indeed, we observed a perturbation of redox signaling, likely owing to PEDV entry. RORγ and NRF2 elicited a much more integrated regulation of the peroxisomes because PEDV infection could limit their physical interaction.

It is documented that although most of the CoVs are unable to hijack the host genetic sequence, they might change the host epigenome [64]. The epigenetic reports proved that RNA viruses antagonize the host epigenome regulatory machine by altering metabolic gene expressions, promoting virus replication and transmission [57,65]. Coincidentally, CoV-2 infection could interact with the host epigenetic machinery to regulate the expression of pro-inflammatory cytokines [66]. In accordance with the notion that H3K4me1 and H3K27ac are the primary modifiers for gene regulation when the host is exposed to CoVs [67], PEDV infection significantly reduced the occupancies of these two histone marks. Furthermore, we also found that the H3K4me2, H3K9ac, and H3K18ac enrichments on *GPX4* and *CAT* were consistently diminished in the livers. These acetylation modifications would be attributed to the p300 loss. This co-activator represents a crucial factor in acetylating CoVs proteins, as reported [68]. As such, it is proven that extended OS produces free radicals to change the epigenetic landscape and p300 binding at target loci, thereby worsening the liver lesions [69,70]. With an emphasis on those direct epigenetic regulations as a consequence of the interaction between PEDV entry and LPO, the other indirect mechanisms are also involved when necessary.

## 5. Conclusions

In conclusion, we have demonstrated that PEDV infection triggers aberrant regulations of anti-oxidative genes *CAT* and *GPX4* in the liver via epigenetic inhibition of ROR/NRF2-mediated transcription, leading to LPO through OS. This might allow us to focus on rebuilding the balance of OS-induced changes during PEDV infection. Given the robust evidence we provided linking changes in histone modifications with PEDV infection, therapeutic strategies targeting bio-modifiers according to these mechanisms may protect the host organs during virus entry. From the perspective of liver physiology, the detailed study of epigenetic regulation will surely be useful for predicting the response to treatment or therapeutic potential of hepatic complications when infected with CoVs.

## Figures and Tables

**Table 1:** Primers for RT-qPCR.

Gene	Primer Sequences (5′ to 3′)
*SLC3A2*	Forward: GCGTCTTCATTCCTGGCTGAGTG
	Reverse: GGATCTGCTGTAGGTCGGAGGAG
*SLC7A11*	Forward: TCTTTGTTGCCCTCTCCTGCTTTG
	Reverse: GAGTGTGTTTGCGGATGTGAATCATG
*GCLM*	Forward: TGTGATGCCGCCCGATTTAACTG
	Reverse: CCACTCATGTGCCTCGATGTCAG
*GCLC*	Forward: AGTTCAACACGGTGGAGGACAATATG
	Reverse: CGGGCAGCCTAATCTGGGAAATG
*GSS*	Forward: GCTGCCAAGATCCTCTCCAATAATCC
	Reverse: CTTGAGCAACCAGTAGCACCAGAG
*GPX4*	Forward: TCCTCATTGATAAGAACGGCTGTGTG
	Reverse: TAGCACGGCAGGTCCTTCTCTATG
*SOD1*	Forward: CTCTCGGGAGACCATTCCATCATTG
	Reverse: TTCTTCATTTCCACCTCTGCCCAAG
*SOD2*	Forward: TTTCTGGACAAATCTGAGCCCTAACG
	Reverse: CGACGGATACAGCGGTCAACTTC
*CAT*	Forward: TGAACGTACTGAATGAGGAGGAGAGG
	Reverse: TCTTGACCGCTTTCTTCTGGATGAAC
*PRX*	Forward: AGGTAGAGGCGACGGAGATGAAAG
	Reverse: TGAGATGGCAAACTTGGACACCTTC
*GAPDH*	Forward: ACATCATCCCTGCTTCTACTGG
	Reverse: CTCGGACGCCTGCTTCAC

**Table 2:** Antibodies for Western blot.

Antibody Name	Supplier	Cat No.
RORγ	eBioscience	12-6988-80
NRF2	Proteintech	66504-1-Ig
*GAPDH*	Huaxing bio	HX1828

**Table 3:** Primers for ChIP-qPCR.

Gene	Primer Sequences (5′ to 3′)
*CAT*	Forward: CCCAGGTTAGATAGGGGTTG
	Reverse: ACGGCTCAGCGGAAATG
*GPX4*	Forward: AACCCACTGAGCAAGGACA
	Reverse: TGACGGGAACTCGCTAAAA
*SOD2*	Forward: AAGTTTTATGTGGTTTCGTTTCCCC
	Reverse: GCAGGAAGTCAACAGGAGCA
*GSS*	Forward: TCTGCAGTTTCATGTTGTCCCATTC
	Reverse: TGGGCGGGAAGAACACT

## Data Availability

The data presented in this study are available in the article.
